# Supplementary material for: NatD promotes lung cancer progression by preventing histone H4 serine phosphorylation to activate Slug expression
Source: Nat Commun. 2017 Oct 13;8:928. doi: 10.1038/s41467-017-00988-5 (PMC5640650; doi:10.1038/s41467-017-00988-5)
Supplement: Supplementary file 1 — Supplementary Information [file 41467_2017_988_MOESM1_ESM.pdf]

Description of Supplementary Files

File Name: Supplementary Information
Description: Supplementary Figures, Supplementary Tables and Supplementary Reference

File Name: Peer Review File

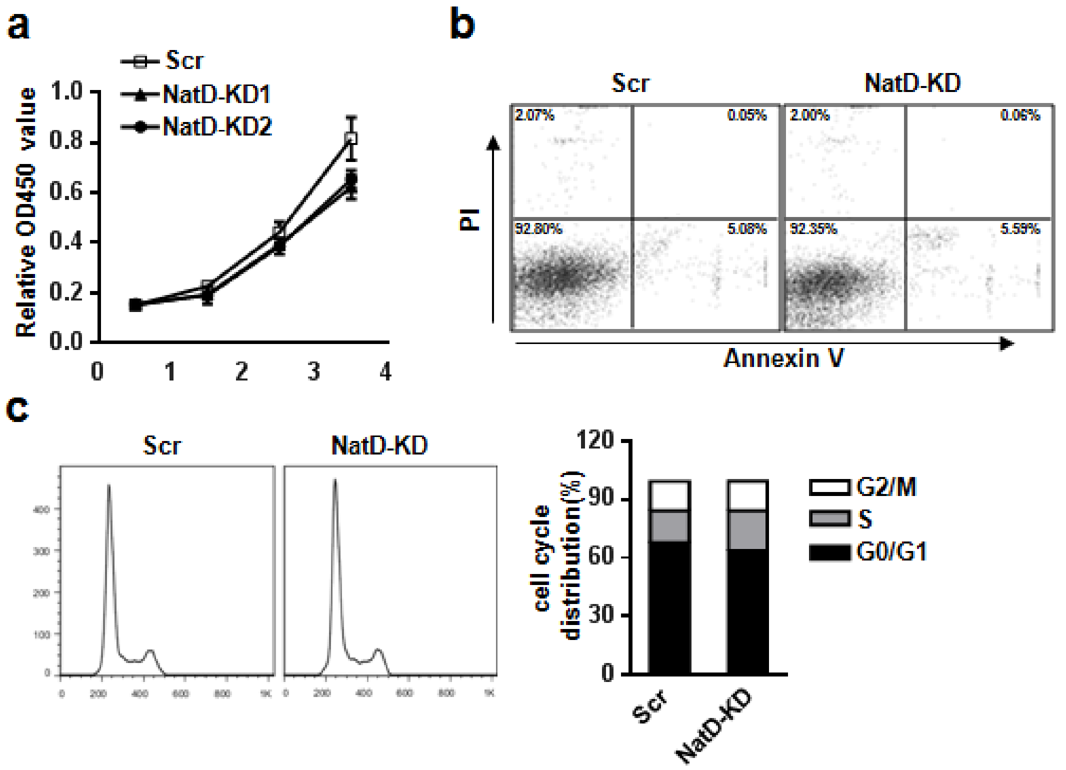

Supplementary Figure 1. Knockdown of NatD has no effect on cell proliferation, apoptosis, or cell cycle in H1299 cells. (**a**) Growth curve of Scr, NatD-KD1, and NatD-KD2 H1299 cells from three independent experiments. (**b** and **c**) Representative images from three independent experiments of apoptosis (**b**) and cell cycle analysis (**c**) of Scr and NatD-KD H1299 cells analyzed by flow cytometry.

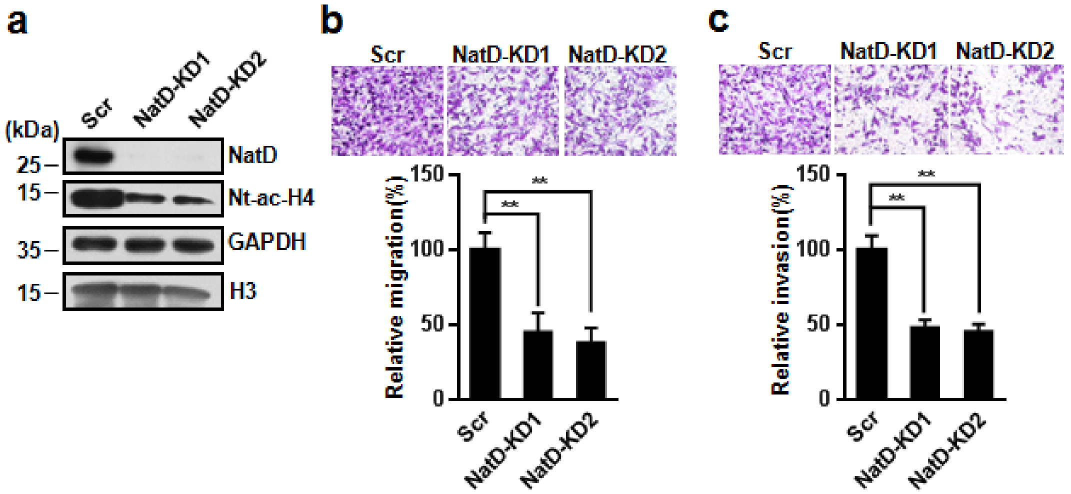

Supplementary Figure 2. NatD knockdown inhibits cell migration and invasion of A549 cells. (**a**) Western blot analysis of NatD and Nt-ac-H4 in Scr, NatD-KD1 and NatD-KD2 A549 cells. Blots are representative of three independent experiments. (**b** and **c**) (top) Images (×100 magnification) from transwell assay of migration (**b**) and invasion (**c**) of Scr, NatD-KD1 and NatD-KD2 A549 cells representative of three independent experiments. (bottom) Quantification of cell migration and invasion expressed as a percentage of Scr control; mean ± s.d. from three independent experiments; two-tailed Student's *t*-test, $^{**}P<0.01$ compared to Scr control.

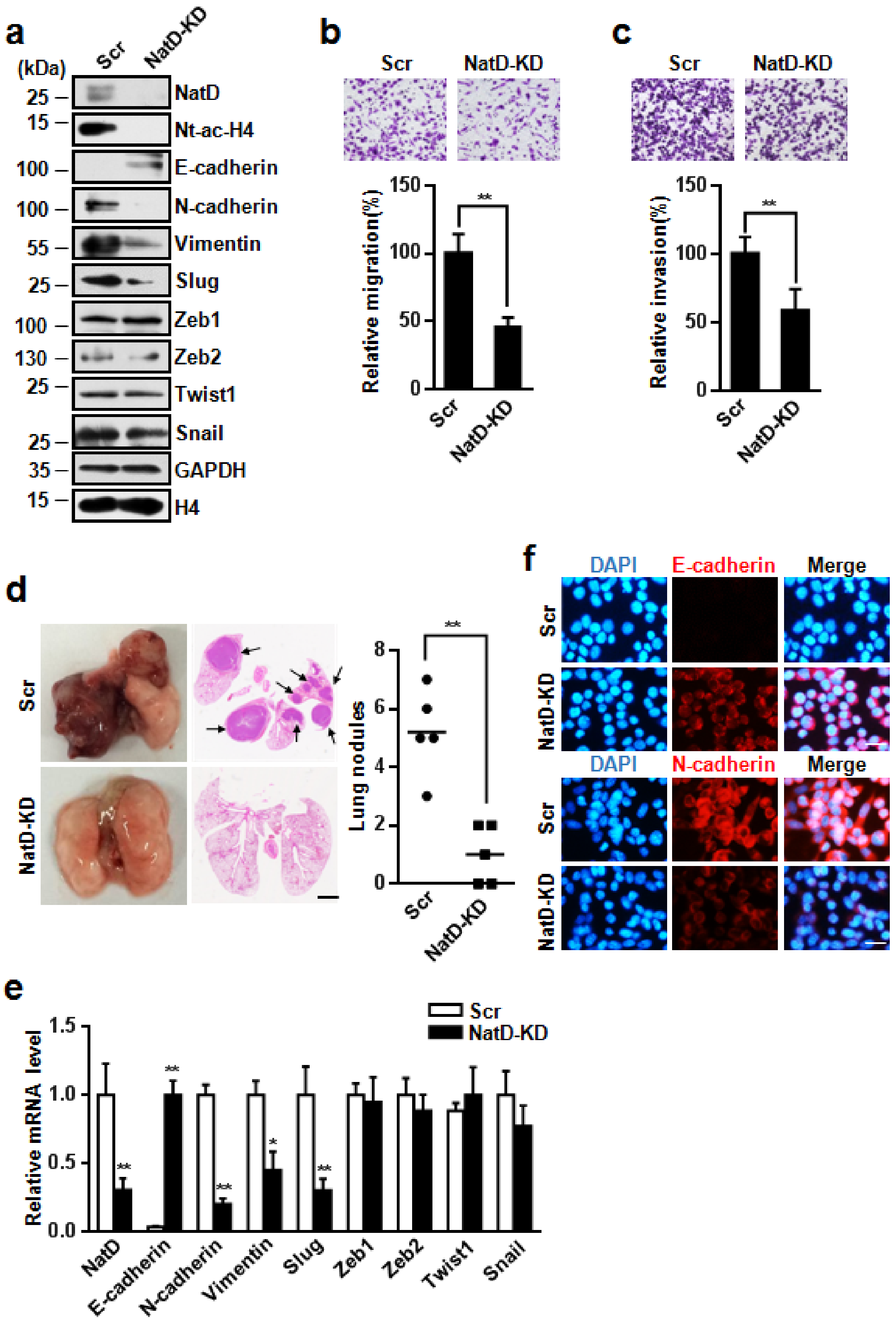

Supplementary Figure 3. NatD knockdown inhibits mouse LLC cell migration and invasion in vivo. (**a**) Western blot analysis of NatD, Nt-ac-H4, and key EMT-

related proteins in Scr control and NatD-KD murine Lewis lung carcinoma (LLC) cells. Human NatD shRNA KD2 targeting sequence (NatD-KD2) was used in mouse. Representative blots from three independent experiments. (**b** and **c**) (top) Representative images of transwell assay of the migration (**b**) and invasion (**c**) of Scr control and NatD-KD LLC cells. Images are representative of three independent experiments. (bottom) Cell migration and invasion expressed as a percentage of Scr control. Calculation based on cell counts for the corresponding assays of at least four random individual fields visualized at ×100 magnification. Results are shown as mean ± s.d. from three independent experiments; two-tailed Student's *t*-test, $^{**}P<0.01$ compared to Scr control. (**d**) (left) Representative images of lung nodules of C57BL/6 mice 30 days after intravenous injection of Scr or NatD-KD LLC cells. (middle) Representative histological images of H&E stained lung from corresponding mice. Scale bars, 2 mm. The arrows indicate major metastatic nodules. (right) Box plot showing numbers of lung nodules from corresponding mice; n=5 mice per group. Results are shown as mean ± s.d. from 5 mice; two-tailed Student's *t*-test, $^{**}P<0.01$ compared to Scr control. (**e**) Quantitative real-time PCR analysis of mRNA levels of NatD and key EMT-related genes in Scr control and NatD-KD LLC cells normalized to GAPDH. Results are shown as mean ± s.d. from three independent experiments; two-tailed Student's *t*-test, $^{*}P<0.05$, $^{**}P<0.01$ compared to Scr control. (**f**)

Immunofluorescence analysis of Scr and NatD-KD LLC cells stained for E-cadherin and N-cadherin. Data are representative of three independent experiments. Scale bar, 20 μm.

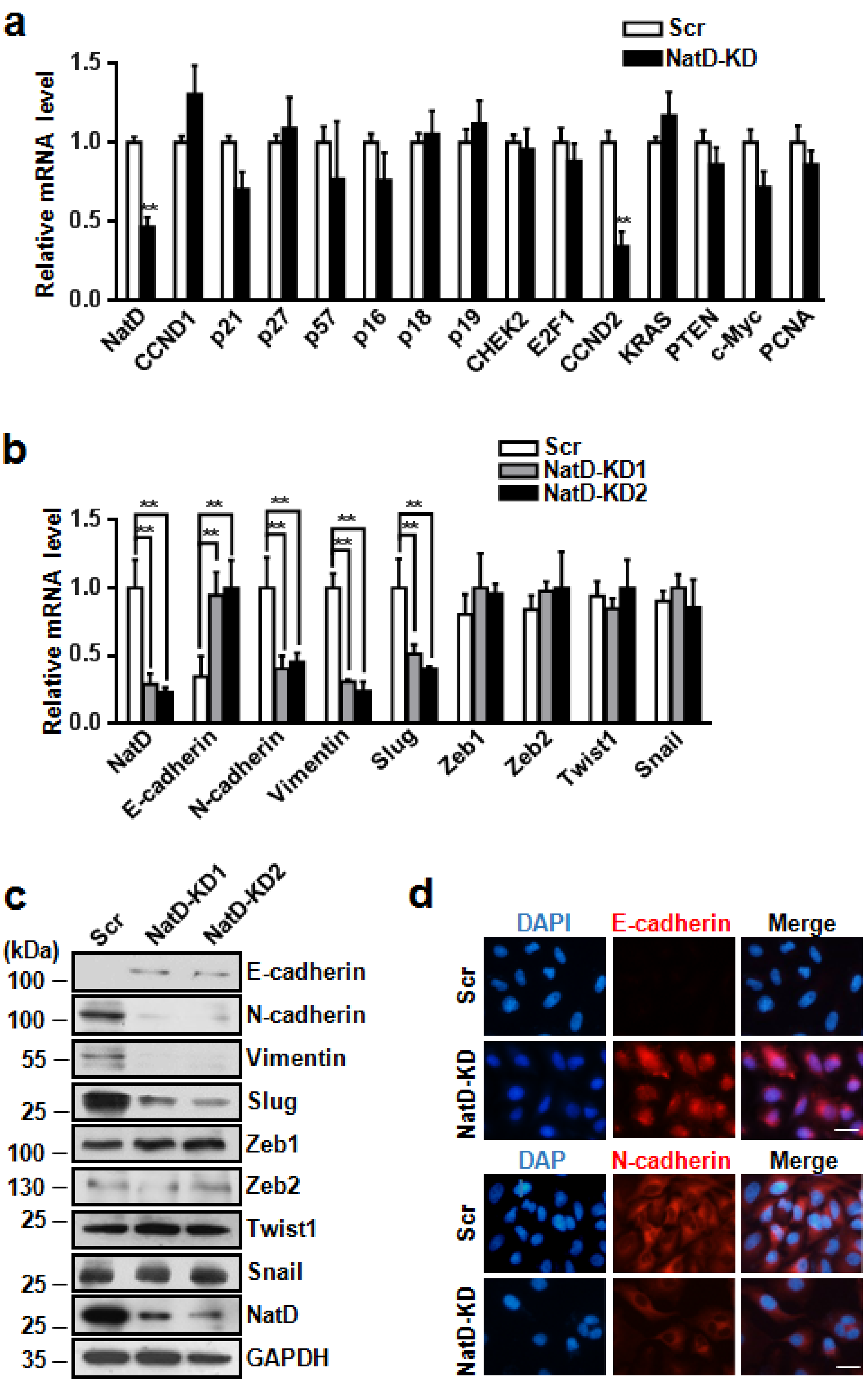

a
Scr
NatD-KD
Relative mRNA level
1.5
1.0
0.5
0.0
**
**
NatD
CCND1
p21
p27
p57
p16
p18
p19
CHEK2
E2F1
CCND2
KRAS
PTEN
c-Myc
PCNA
b
Scr
NatD-KD1
NatD-KD2
Relative mRNA level
1.5
1.0
0.5
0.0
NatD
E-cadherin
N-cadherin
Vimentin
Slug
Zeb1
Zeb2
Twist1
Snail
c
Scr
NatD-KD1
NatD-KD2
(kDa)
100
100
55
25
100
130
25
25
25
35
E-cadherin
N-cadherin
Vimentin
Slug
Zeb1
Zeb2
Twist1
Snail
NatD
GAPDH
d
DAPI
E-cadherin
Merge
Scr
NatD-KD
DAP
N-cadherin
Merge
Scr
NatD-KD

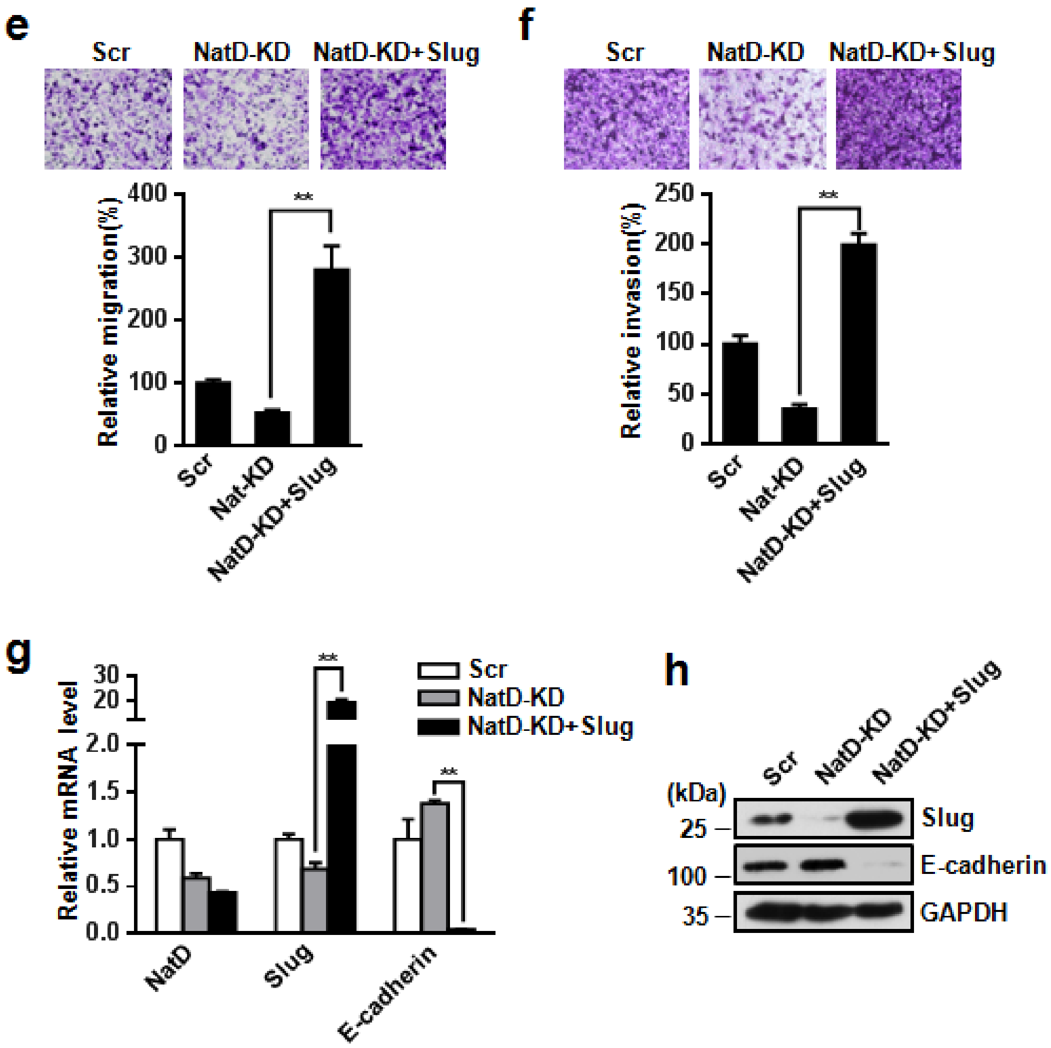

Supplementary Figure 4. NatD knockdown suppresses cell migration and invasion by downregulating Slug in A549 cells. (**a**) Quantitative real-time PCR analysis of mRNA levels of NatD, CCND1, p21, p27, p57, p16, p18, p19, CHEK2, E2F1, CCND2, KRAS, PTEN, c-Myc, and PCNA in scrambled (Scr) and NatD knockdown (NatD-KD) H1299 cells normalized to GAPDH. Results are shown as mean ± s.d. of three independent experiments; two-tailed Student's *t*-test, $**P<0.01$ compared to Scr control. (**b**) Quantitative real-time PCR analysis of mRNA levels of NatD and key EMT-related genes in Scr and NatD-KD A549 cells normalized to

GAPDH. Results are shown as mean ± s.d. of three independent experiments; two-tailed Student's *t*-test, $^{**}P<0.01$ compared to Scr control. (**c**) Western blot analysis of expression of NatD and key EMT-related proteins in Scr and NatD-KD A549 cells. GAPDH served as a loading control. Blots are representative of three independent experiments. (**d**) Immunofluorescence analysis of Scr and NatD-KD A549 cells stained for E-cadherin and N-cadherin. Data are representative of three independent experiments. Scale bar, 20 μm. (**e** and **f**) (top) Representative images of transwell assay of migration (**e**) and invasion (**f**) of scrambled (Scr), NatD knockdown (NatD-KD) and Slug-expressing NatD-KD (NatD-KD+Slug) A549 cells from three independent experiments. (bottom) Cell migration and invasion expressed as a percentage of control. Calculations based on cell counts from the corresponding assays of at least four random fields at ×100 magnification from three independent experiments. Results are shown as mean ± s.d. of three independent experiments; two-tailed Student's *t*-test, $^{**}P<0.01$ compared to indicated control. (**g**) Quantitative real-time PCR analysis of the mRNA levels of NatD, Slug, and E-cadherin normalized to GAPDH in Scr, NatD-KD, and Slug-expressing NatD-KD A549 cells. Results are shown as mean ± s.d. of three independent experiments; two-tailed Student's *t*-test, $^{**}P<0.01$ compared to indicated control. (**h**) Western blot analysis of Slug and E-cadherin in scrambled, NatD-KD, and Slug- expressing NatD-KD A549 cells.

GAPDH served as a loading control. Blots are representative of three independent experiments.

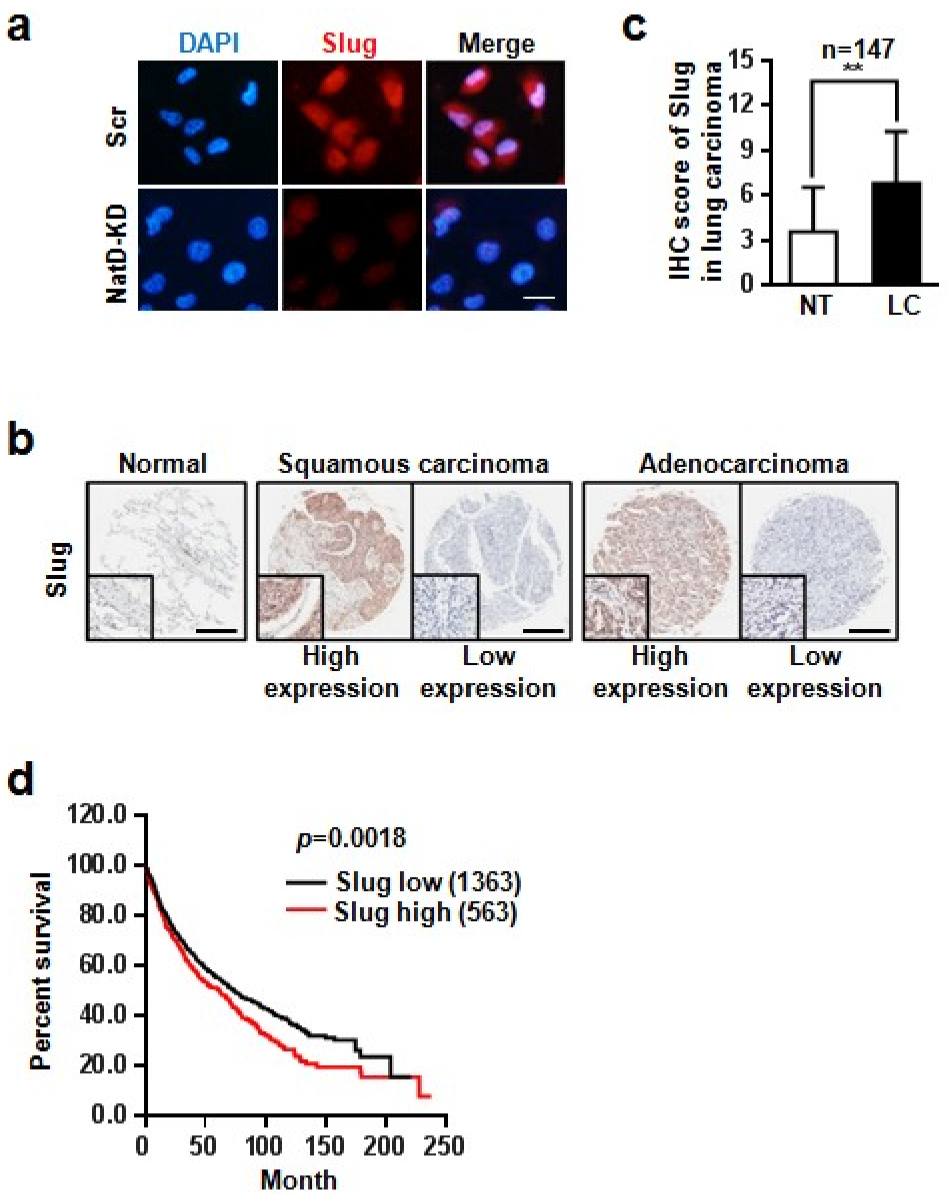

Supplementary Figure 5. Slug expression in human lung cancer tissues. (a) Immunofluorescence analysis of Scr and NatD-KD H1299 cells stained for Slug. Data are representative of three independent experiments. Scale bar, 20 μm. (**b**) Representative images of immunohistochemical (IHC) staining of Slug in normal tissues (n=147), human lung squamous carcinoma (n=74), and lung adenocarcinoma (n=73) tissue samples. Scale bars, 500 μm. (**c**) Total IHC score of Slug in matched

normal tissues (NT) and lung carcinoma (LC); mean ± s.d. of 147 pairs of tissue samples. two-tailed Student's *t*-test, $^{**}P<0.01$ compared to matched normal tissue control. (**d**) Kaplan–Meier plots of overall survival of patients with lung cancer, stratified by Slug expression. Data were obtained from Kaplan-Meier plotter database; log-rank test, P=0.0018.

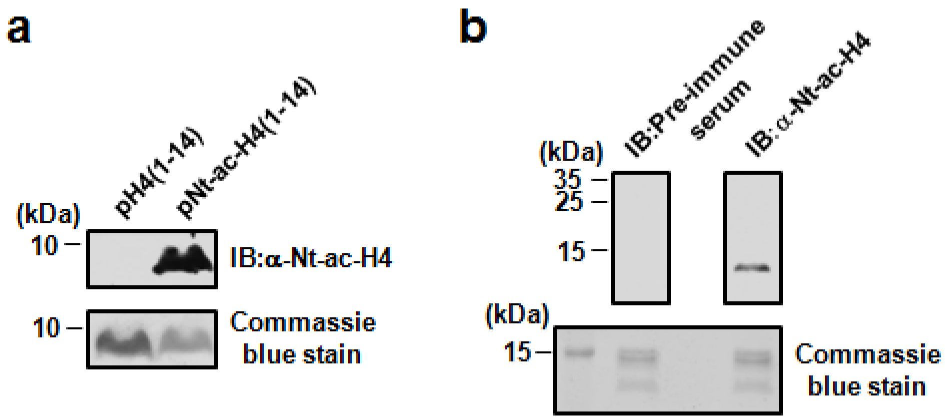

Supplementary Figure 6. Validation of anti-Nt-ac-H4 antibody. (**a**) Western blot analysis of acetylated (pNt-ac-H4) or non-acetylated (pH4) peptides with anti-Nt-ac-H4 antibody. (**b**) (top) Western blot analysis of histones from H1299 cells with pre-immune serum or with anti-Nt-ac-H4 antibody. Coomassie blue staining of histones isolated from H1299 cells (bottom panel). Blots in (**a**) and (**b**) are representative of three independent experiments.

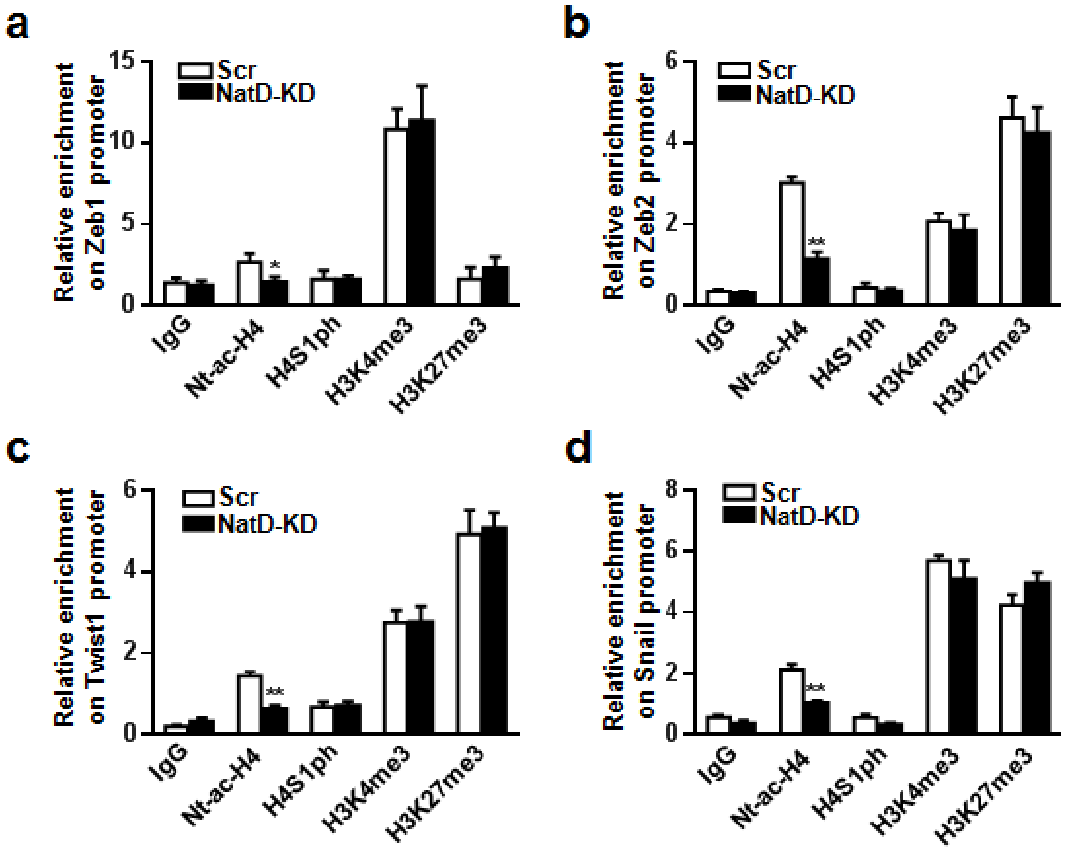

Supplementary Figure 7. Knockdown of NatD has no effect on enrichment of H4S1ph on some EMT-related gene promoters. (**a**-**d**) ChIP analysis of the enrichment of Nt-ac-H4, H4S1ph, H3K4me3 and H3K27me3 on the promoter of Zeb1(**a**), Zeb2(**b**), Twist1(**c**), and Snail (**d**) in Scr and NatD-KD H1299 cells. IgG served as a negative control. Results are shown as mean ± s.d. of three independent experiments; two-tailed Student's *t*-test, $^{**}P<0.01$ or $^{*}P<0.05$ compared to Scr control.

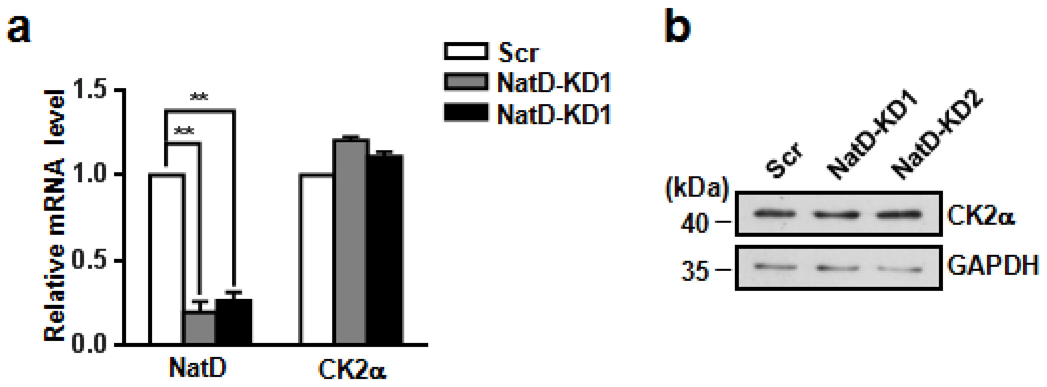

Supplementary Figure 8. Knockdown of NatD has no effect on expression of CK2α. (**a**) Quantitative real-time PCR analysis of NatD and CK2α mRNA levels in scrambled and NatD-KD H1299 cells normalized to GAPDH. Results are shown as mean ± s.d. of three independent experiments; two-tailed Student's *t*-test, $^{**}P<0.01$ compared to Scr control. (**b**) Western blot analysis of CK2α protein in Scr and NatD-KD H1299 cells. GAPDH served as a loading control. Blots are representative of three independent experiments.

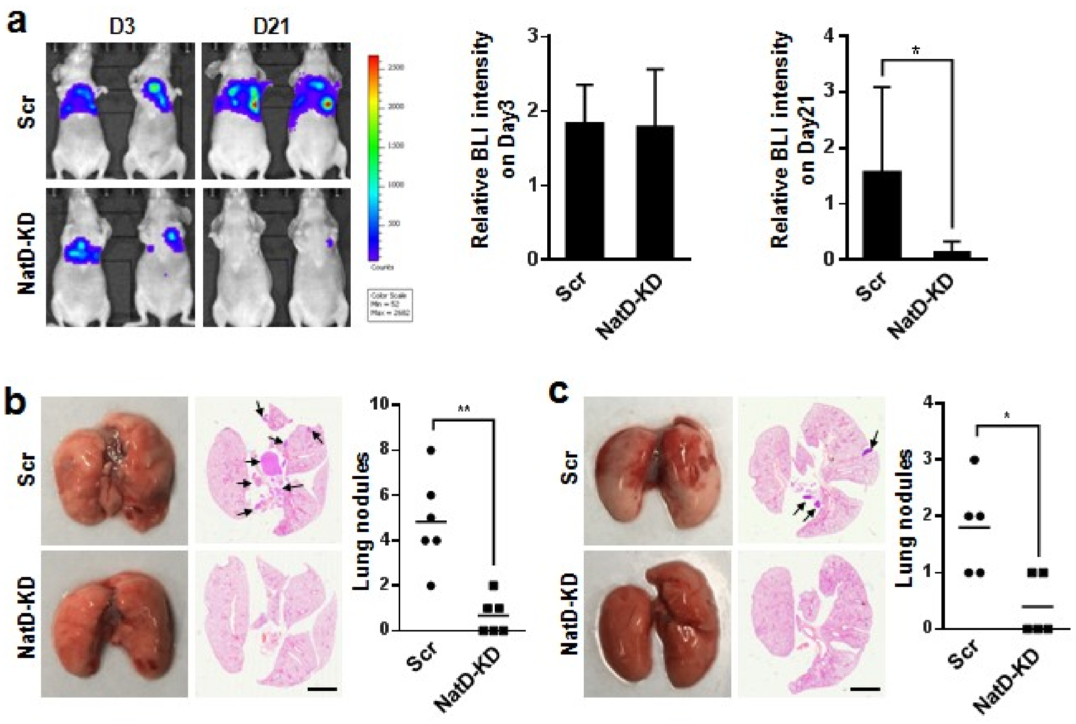

Supplementary Figure 9. NatD knockdown inhibits the migration and invasion of human A549 cells and murine LLC cells in orthotopic lung tumor implantation models. (**a**) (left) Representative bioluminescent (BLI) images acquired at the indicated time points after orthotopic injection with luciferase-labeled Scr or NatD-KD A549 cells in nude mice. Pseudocolor heat-maps indicate intensity of bioluminescence from low (blue) to high (red) (D, day). (right) Relative BLI signals of lung tumors of corresponding mice (n = 6 for each group) recorded on day 3 and day 21. Results are shown as mean ± s.d. from 6 mice. Two-tailed Student's t-test was used. **P*<0.05 compared to Scr control. (**b**) (left) Representative images of lung nodules in nude mice acquired 21 days after orthotopic injection with Scr or NatD-KD A549 cells. (middle) Representative images of H&E stained histological sections

of lungs from nude mice. Scale bars, 2 mm. Arrows indicate major lung nodules. (right) Box plot showing numbers of lung nodules from corresponding mice (n = 6 for each group). Results are shown as mean ± s.d. from 6 mice. Two-tailed Student's *t*-test was used. **P*<0.05 compared to Scr control. (**c**) (left) Representative images of lung nodules of C57BL/6 mice 14 days after orthotopic injection of Scr or NatD-KD LLC cells. (middle) Representative histological images of H&E stained lung from corresponding mice. Scale bars, 2 mm. The arrows indicate major metastatic nodules. (right) Box plot showing numbers of lung nodules from corresponding mice (n = 5 mice per group).

Supplementary Figure 10. Original images of Western blot

Figure 2b NatD

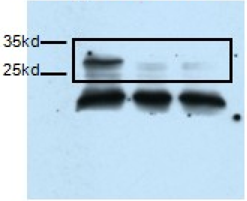

Figure 2b Nt-ac-H4

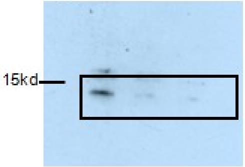

Figure 2b GAPDH

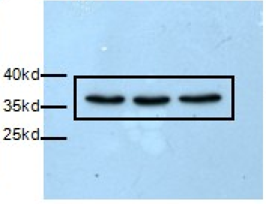

Figure 2b H4

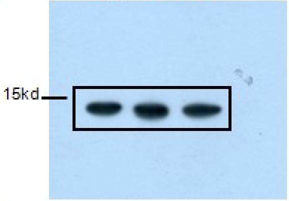

Figure 4c E-cadherin

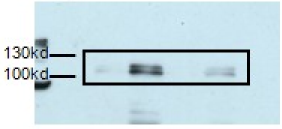

Figure 4c N-cadherin

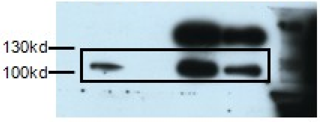

Figure 4c Vimentin

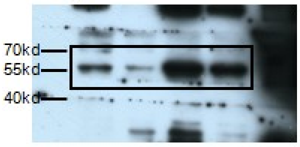

Figure 4c Slug

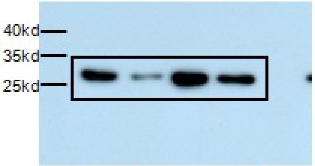

Figure 4c Zeb1

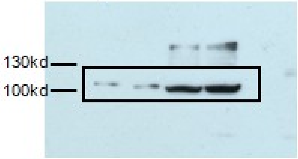

Figure 4c Zeb2

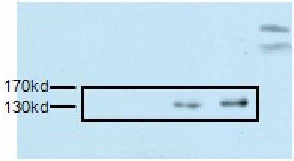

Figure 4c Twist1

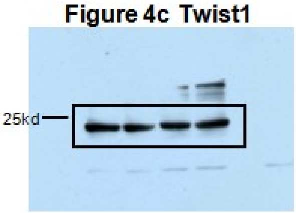

Figure 4c Snail

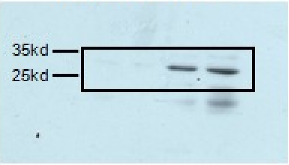

Figure 4c NatD

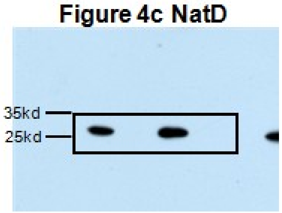

Figure 4c GAPDH

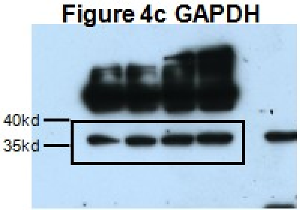

Figure 4h Slug

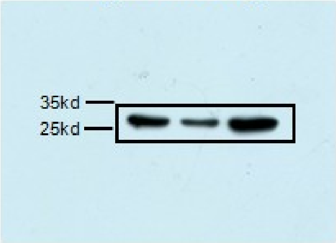

Figure 4h E-cadherin

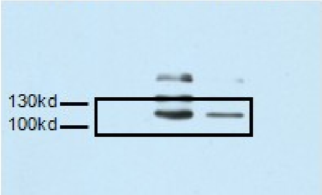

Figure 4h N-cadherin

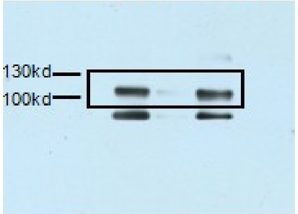

Figure 4h Vimentin

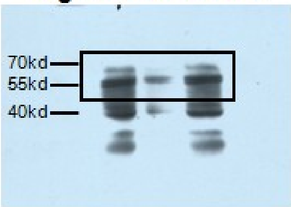

Figure 4h Zeb1

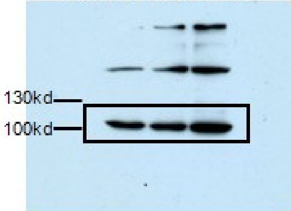

Figure 4h Zeb2

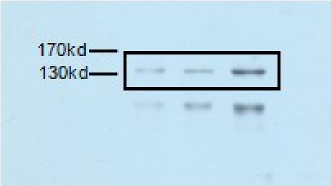

Figure 4h Twist1

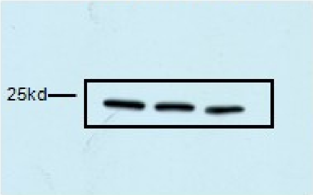

Figure 4h Snail

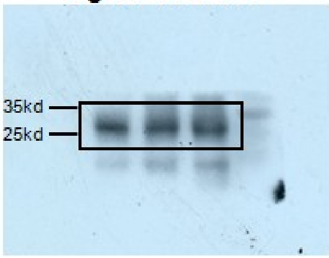

Figure 4h GAPDH

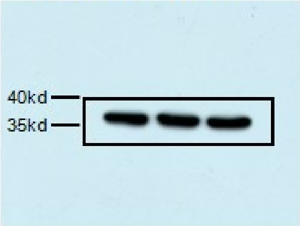

Figure 5b Nt-ac-H4

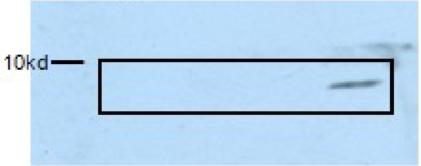

Figure 5c autoradiography

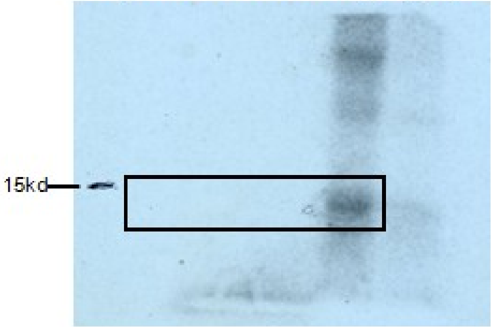

Figure 5e Slug

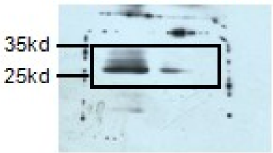

Figure 5e E-cadherin

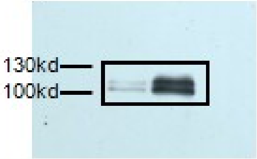

Figure 5e N-cadherin

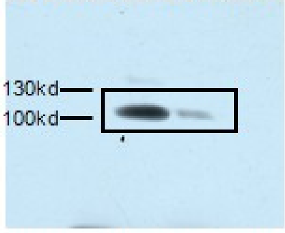

Figure 5e Vimentin

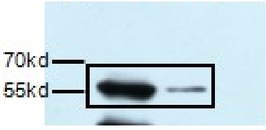

Figure 5e NatD

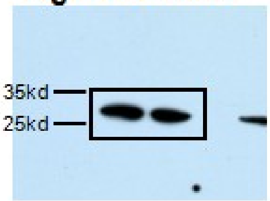

Figure 5e Nt-ac-H4

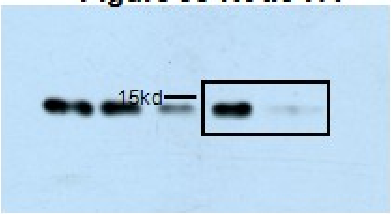

Figure 5e GAPDH

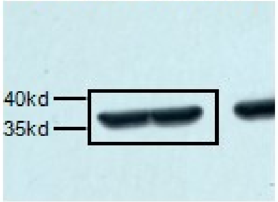

Figure 5e H4

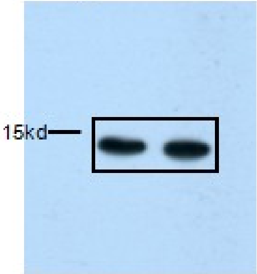

Figure 6a Nt-ac-H4

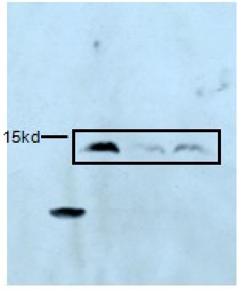

Figure 6a H4S1ph

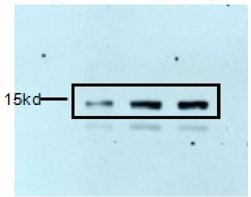

Figure 6a H4R3me2a

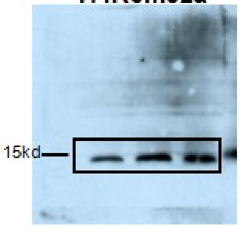

Figure 6a H4R3me2s

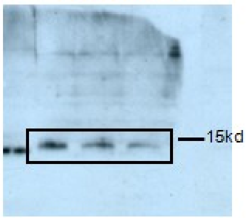

Figure 6a H4K5ac

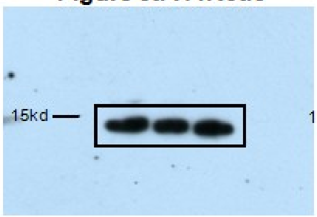

Figure 6a H4K8ac

Figure 6a H4K12ac

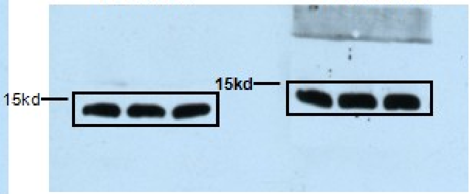

Figure 6a H4

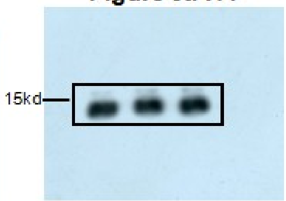

Figure 6d Nt-ac-H4

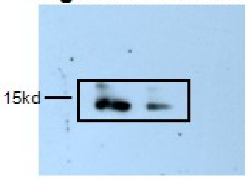

Figure 6d H4S1ph

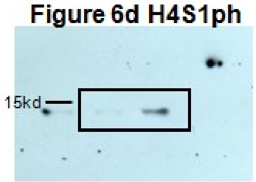

Figure 6d Slug

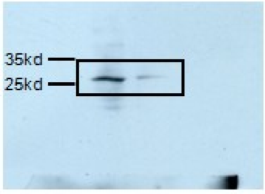

Figure 6d NatD

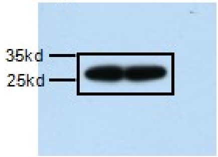

Figure 6d GAPDH

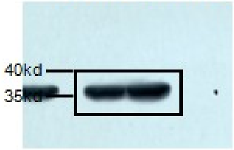

Figure 6d H4

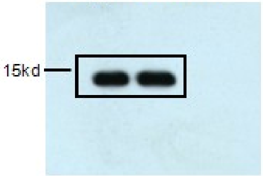

Figure 7b CK2α

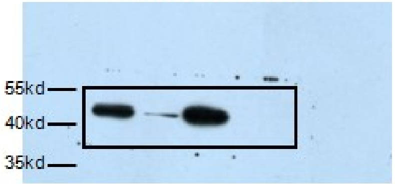

Figure 7b Lamin A/C

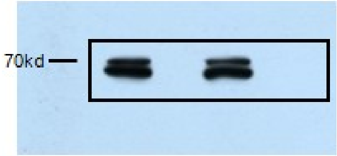

Figure 7b HSP70

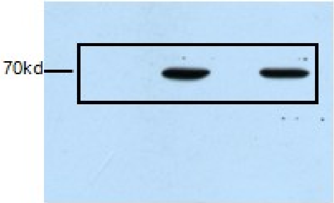

Figure 7c CK2α

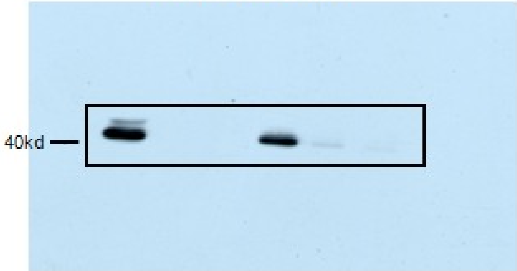

Figure 7c Streptavidin

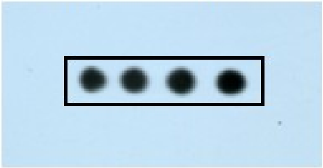

Figure 7c Nt-ac-H4

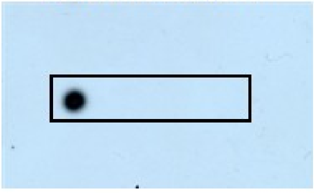

Figure 7h Slug

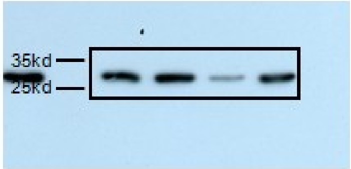

Figure 7h CK2α

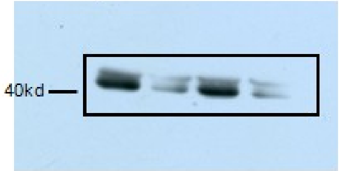

Figure 7h GAPDH

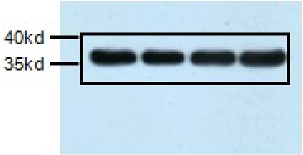

Supplemental Figure 2a NatD

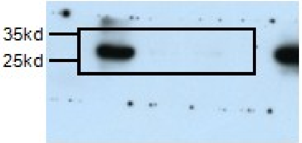

Supplemental Figure 2a Nt-ac-H4

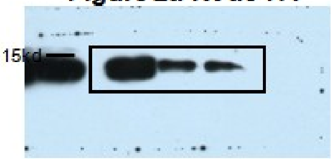

Supplemental Figure 2a GAPDH

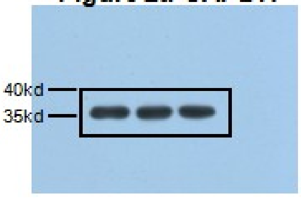

Supplemental Figure 2a H3

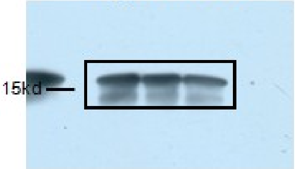

Supplemental Figure 3a NatD

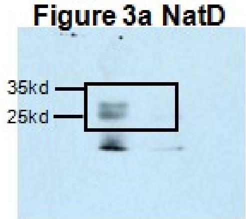

Supplemental Figure 3a Nt-ac-H4

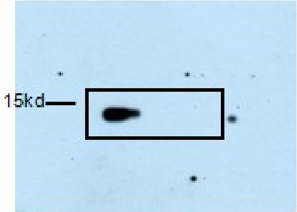

Supplemental Figure 3a E-cadherin

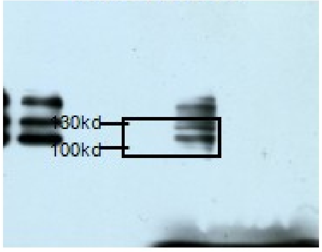

Supplemental Figure 3a N-cadherin

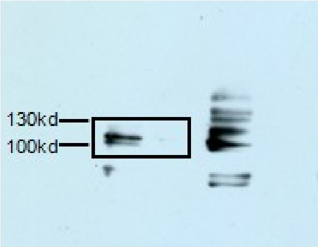

Supplemental Figure 3a Vimentin

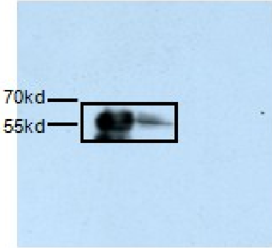

Supplemental Figure 3a Slug

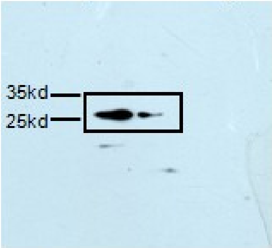

Supplemental Figure 3a Zeb1

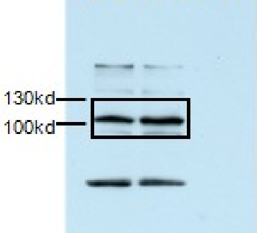

Supplemental Figure 3a Zeb2

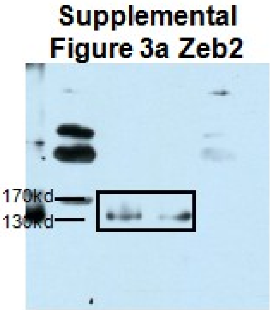

Supplemental Figure 3a Twist1

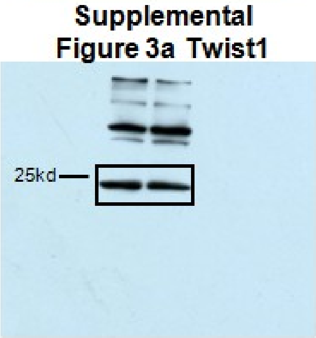

Supplemental Figure 3a Snail

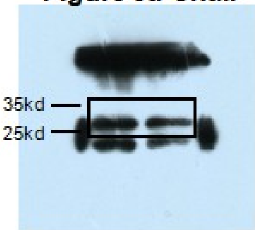

Supplemental Figure 3a GAPDH

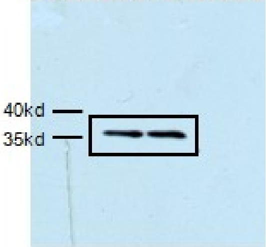

Supplemental Figure 3a H4

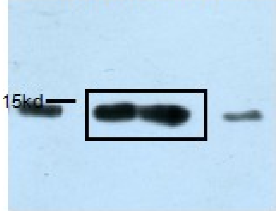

Supplemental Figure 4c E-cadherin

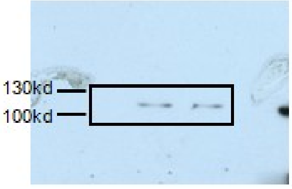

Supplemental Figure 4c N-cadherin

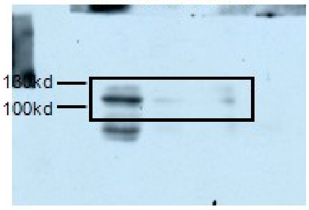

Supplemental Figure 4c Vimentin

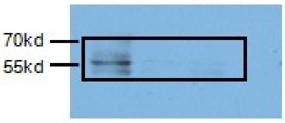

Supplemental Figure 4c Slug

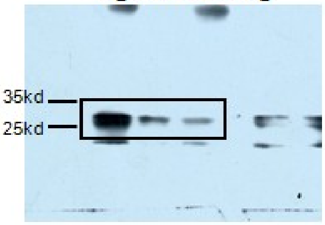

Supplemental Figure 4c Zeb1

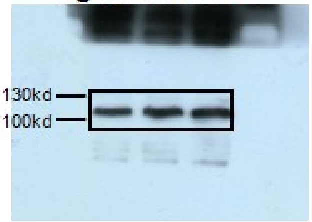

Supplemental Figure 4c Zeb1

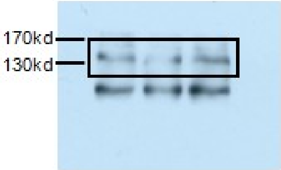

Supplemental Figure 4c Twist1

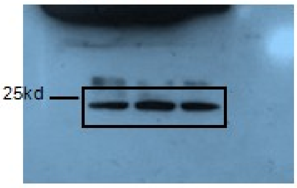

Supplemental Figure 4c Snail

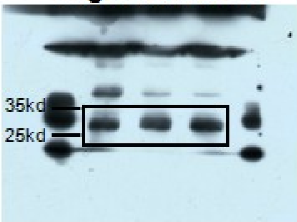

Supplemental Figure 4c NatD

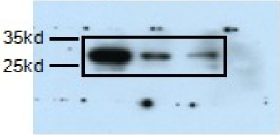

Supplemental Figure 4c GAPDH

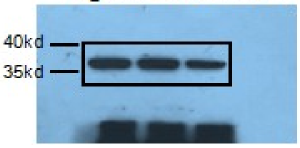

Supplemental Figure 4h Slug

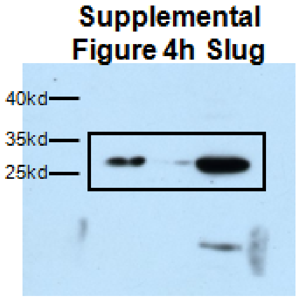

Supplemental Figure 4h E-cadherin

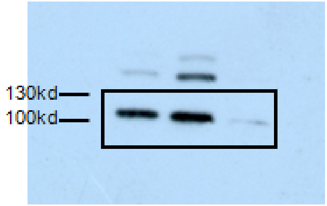

Supplemental Figure 4h GAPDH

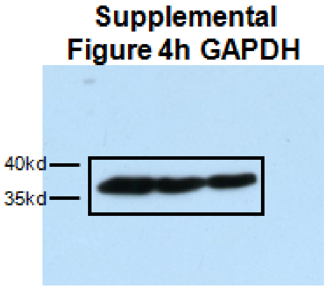

Supplemental Figure 6a Commassie blue stain

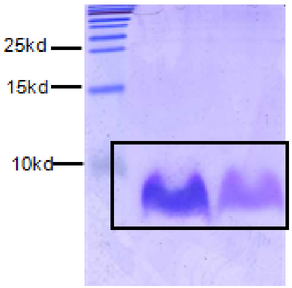

Supplemental Figure 6a Nt-ac-H4

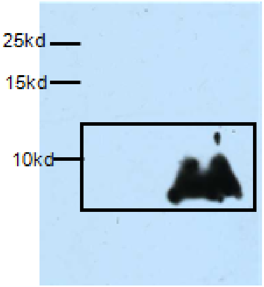

Supplemental Figure 6b Nt-ac-H4

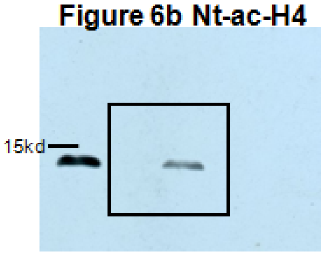

Supplemental Figure 8b CK2α

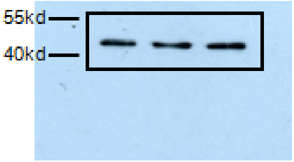

Supplemental Figure 8b GAPDH

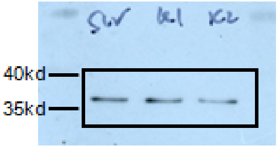

Supplementary Table 1: Clinicopathologic characteristics of NatD expression in lung cancer patients

Characteristics	Cases	IHC score (Mean ± s.d.)	*P* value[a]
	147		
Gender			*0.1097*
Male	109	7.303±3.604	
Female	38	8.421±3.922	
Age			*0.3480*
>60	81	7.852±3.818	
≤60	66	7.273±3.571	
Tumor size			*0.0182*
≥5 cm	58	8.483±3.440	
<5 cm	89	7.011±3.780	
Tumor stage [b]			*0.6421*
I-II	104	7.500±3.642	
III	43	7.814±3.899	
Lymph node status [b]			*0.0367*
N0	82	7.024±3.641	
N1-3	65	8.308±3.695	
Distant metastasis [b]			*0.7345*
M0	138	7.565±3.733	
M1	9	8.000±3.465	
Histology			*0.1332*
Adenocarcinoma	73	8.055±3.858	
Squamous carcinoma	74	7.135±3.520	

[a]*P* values were derived using two-sided Pearson χ^2 tests to compare values for the two parameters in each category.

[b]The tumor stage, lymph node status, and metastasis were classified according to the international system for staging lung cancer[1].

Supplementary Table 2. Real-time PCR primer sequences (human)

Locus	Forward	Reverse
NATD:	ATGTAAGCGAGTGTCTGGACT	TGGTTTGCATATTCGTTTTGGTC
Slug:	CTGTGACAAGGAATATGTGAGCC	CAAATGCTCTGTTGCAGTGAG
Twist1:	GGAGTCCGCAGTCTTACGAG	TCTGGAGGACCTGGTAGAGG
N-cadherin:	CCCTGCTTCAGGCGTCTGTA	TGCTTGCATAATGCGATTTCACC
Vimentin:	CCACCAGGTCCGTGTCCTCGT	CGCTGCCCAGGCTGTAGGTG
E-cadherin:	TTGCACCGGTCGACAAAGGAC	TGGAGTCCCAGGCGTAGACCAA
Snail:	GGAAGCCTAACTACAGCGAGCT	CTGGAAGGTAAACTCTGGATTAG
Zeb1	GGTCATGATGAAAATGGAACACC	AGGTGTAACTGCACAGGGAGCA
Zeb2	GACAGATCAGCACCAAATGC	GCTGATGTGCGAACTGTAGG
CCND1:	CAATGACCCCGCACGATTTC	CATGGAGGGCGGATTGGAA
CCND2:	ACCTTCCGCAGTGCTCCTA	CCCAGCCAAGAAACGGTCC
p21:	CTGGAGACTCTCAGGGTCGAAA	GATTAGGGCTTCCTCTTGGAG
p27:	GGAGCAATGCGCAGGAATAA	TGGGGAACCGTCTGAAACAT
p57:	GCGTCCCTCCGCAGCACAT	GGTTCTGGTCCTCGGCGTTCA
p16:	CCCCTTGCCTGGAAAGATAC	AGCCCCTCCTCTTTCTTCCT
p18:	ACTGGTTTCGCTGTCATTCA	GCAGGTTCCCTTCATTATCC
p19:	ATGCTGCTGGAGGAGGTTCG	GTCTTGCCGAAGCGGTTGAG
E2F1:	AGCTGGACCACCTGATGAAT	GAGGGGCTTTGATCACCATA
CHEK2:	TCTCGGGAGTCGGATGTTGAG	CCTGAGTGGACACTGTCTCTAA
KRAS:	AGTGCCTTGACGATACAGC	ACAAAGAAAGCCCTCCC
PCNA:	ACACTAAGGGCCGAAGATAACG	ACAGCATCTCCAATATGGCTGA
c-Myc	CAGCTGCTTAGACGCTGGATTT	ACCGAGTCGTAGTCGAGGTCAT
PTEN	TTTGAAGACCATAACCCACCAC	ATTACACCAGTTCGTCCCTTTC
GAPDH	GAAGGTGAAGGTCGGAG	GAAGATGGTGATGGGATTTC

Supplementary Table 3. Real-time PCR primer sequences (mouse)

Locus	Forward	Reverse
NatD	GGGGAGAAAGTCGAGCAAAG	CCCATTGCGGTCATACTTCTTG
E-cadherin	CAGTTCCGAGGTCTACACCTT	TGAATCGGGAGTCTTCCGAAAA
N-cadherin	ATAGCCCGGTTTCACTTGAGA	CAGGCTTTGATCCCTCTGGA
Slug	CAGCGAACTGGACACACACA	ATAGGGCTGTATGCTCCCGAG
Vimentin	TCCACACGCACCTACAGTCT	CCGAGGACCGGGTCACATA
Zeb1	ACTGCAAGAAACGGTTTTCCC	GGCGAGGAACACTGAGATGT
Zeb2	ATTGCACATCAGACTTTGAGGAA	ATAATGGCCGTGTCGCTTCG
Twist1	TGCAGGACGTGTCCAGCTC	CTGCTGCGTCTCTTGCGAG
Snail	CACACGCTGCCTTGTGTCT	GGTCAGCAAAAGCACGGTT
GAPDH	AGGTCGGTGTGAACGGATTTG	GGGGTCGTTGATGGCAACA

Supplementary Table 4. ChIP primer sequences

Locus	Forward	Reverse
Slug:	CTGAACCTCTCAGCTGTGATTGG	CTTTACGAACTGAGCCCGTTTT
Snail	AAAAAGGCCGTGGCATTTCAA	GGGACACCTGACCTTCCGAC
Zeb1	AAGTCACTTCCCATCCCGGTT	CCAAGGAAAGGGATCGCGG
Zeb2	TGTAGTGAGGTCTCCCCGAG	TGCATGGGAGCTGCATCTTA
Twist1	CTCCAAGGGGTTCGTCTACC	ACAGCTTCTACACAGTGGGTG

Supplementary Reference:

1. Mountain, C. F. The international system for staging lung cancer. *Semin. Surg. Oncol*. **18**:106-15 (2000).
